# A Model System for *In Vitro* Studies of Bank Vole Borne Viruses

**DOI:** 10.1371/journal.pone.0028992

**Published:** 2011-12-16

**Authors:** Malin Stoltz, Karin B. Sundström, Åsa Hidmark, Conny Tolf, Sirkka Vene, Clas Ahlm, A. Michael Lindberg, Åke Lundkvist, Jonas Klingström

**Affiliations:** 1 Department of Microbiology, Tumor and Cell Biology, Karolinska Institutet, Stockholm, Sweden; 2 Department of Preparedness, Swedish Institute for Communicable Disease Control, Solna, Sweden; 3 Department of Infectious Diseases, Medical Microbiology and Hygiene, University of Heidelberg, Heidelberg, Germany; 4 School of Natural Sciences, Linnaeus University, Kalmar, Sweden; 5 Department of Clinical Microbiology, Infectious Diseases, Umeå University, Umeå, Sweden; 6 Department of Epidemiology, Swedish Institute for Communicable Disease Control, Solna, Sweden; Johns Hopkins University - Bloomberg School of Public Health, United States of America

## Abstract

The bank vole (*Myodes glareolus*) is a common small mammal in Europe and a natural host for several important emerging zoonotic viruses, e.g. Puumala hantavirus (PUUV) that causes hemorrhagic fever with renal syndrome (HFRS). Hantaviruses are known to interfere with several signaling pathways in infected human cells, and HFRS is considered an immune-mediated disease. There is no *in vitro*-model available for infectious experiments in bank vole cells, nor tools for analyses of bank vole immune activation and responses. Consequently, it is not known if there are any differences in the regulation of virus induced responses in humans compared to natural hosts during infection. We here present an *in vitro*-model for studies of bank vole borne viruses and their interactions with natural host cell innate immune responses. Bank vole embryonic fibroblasts (VEFs) were isolated and shown to be susceptible for PUUV-infection, including a wild-type PUUV strain (only passaged in bank voles). The significance of VEFs as a model system for bank vole associated viruses was further established by infection studies showing that these cells are also susceptible to tick borne encephalitis, cowpox and Ljungan virus. The genes encoding bank vole IFN-β and Mx2 were partially sequenced and protocols for semi-quantitative RT-PCR were developed. Interestingly, PUUV did not induce an increased IFN-β or Mx2 mRNA expression. Corresponding infections with CPXV and LV induced IFN-β but not Mx2, while TBEV induced both IFN-β and Mx2.

In conclusion, VEFs together with protocols developed for detection of bank vole innate immune activation provide valuable tools for future studies of how PUUV and other zoonotic viruses affect cells derived from bank voles compared to human cells. Notably, wild-type PUUV which has been difficult to cultivate *in vitro* readily infected VEFs, suggesting that embryonic fibroblasts from natural hosts might be valuable for isolation of wild-type hantaviruses.

## Introduction

Bank voles (*Myodes glareolus*) are found in most areas of Europe and in parts of Northern Asia [1]. This rodent is a natural host for several viruses with different characteristics, including the hantavirus Puumala virus (PUUV), the flavivirus tick borne encephalitis virus (TBEV), the orthopoxvirus cowpox virus (CPXV), and the parechovirus Ljungan virus (LV) [2]–[5]. As for many zoonotic agents, PUUV, TBEV and CPXV normally cause asymptomatic infections in their natural host. However, in humans PUUV and TBEV infection may cause severe illness (Table 1). If LV infections cause disease in humans and/or in bank voles is controversial and remains to be clearly shown [6].

**Table 1:** Summary of bank vole borne viruses pathogenic to humans.

	Virus		
	PUUV	TBEV	CPVX
Disease in humans	HFRS	Encephalitis	Mild skin lesions
Disease in rodent host	No	No	No

CPXV, Cowpox virus; HFRS, hemorrhagic fever with renal syndrome; TBEV, tick-borne encephalitis virus, PUUV, Puumala virus.

Rodent borne hantaviruses are the etiological agent of two zoonotic diseases: hemorrhagic fever with renal syndrome (HFRS) in Eurasia, and hantavirus cardiopulmonary syndrome (HCPS) in the Americas. The bank vole borne PUUV causes HFRS in Europe [3]. Currently no specific treatment or US FDA-approved vaccines are available against HFRS/HCPS. While it has been shown that hantaviruses can deregulate human endothelial cell functions [7], activate unusual immune responses in patients [2], [8], and interfere with several signaling pathways in human cells [9] the exact mechanism of symptoms associated with HFRS/HCPS still remains unknown. Interestingly, while hantaviruses cause a transient infection and disease in humans, infections of the natural rodent hosts seem to be chronic and asymptomatic. It is possible that PUUV and other hantaviruses have developed features that facilitate viral persistence, replication and spread, without harming the natural host [10]. However, due to the lack of specific reagents, it has not been possible to analyze if PUUV interferes with bank vole cell functions, and consequently it is not known if PUUV affects infected cells in a species-specific manner.

Activation of innate immune responses is crucial in order to eradicate virus infections. The induction of, as well as response to, type I IFNs (IFN-α/β) during virus infection are important parts of the innate immunity [11]. When pattern recognition receptors (PRR) detect viruses in infected cells, they activate pathways leading to the induction and production of IFN-α/β. These IFNs then act on infected as well as non-infected bystander cells by inducing IFN-stimulated genes (ISGs) like Mx proteins, which possess antiviral features, thereby inhibiting viral replication and further spread. Consequently, most pathogenic viruses have evolved countermeasures which can inhibit induction of IFNs, and/or can inhibit IFN-induced activation of antiviral responses in infected cells [12], [13].

Understanding how zoonotic virus infections in natural hosts differ from human infections might give important clues to the mechanisms behind pathogenesis in humans. In order to study this there is a need for *in vitro*-models based on natural hosts cells. Here, we present a model for virus infection experiments in cells derived from bank voles and protocols for studies of bank vole innate immune activation.

## Materials and Methods

### Ethics statement

Adult bank voles were housed in the Astrid Fagraeus laboratory at the Swedish Institute for Communicable Disease Control. Housing and care procedures were in compliance with the provisions and general guidelines of the Swedish Animal Welfare Agency. Voles were housed following a 12-h light-dark cycle and were provided food and water *ad libitum*. Ethical permission was approved by the Northern Stockholm's animal research ethics committee (Stockholms norra djurförsöksetiska nämnd, permit numbers N33/06 and N106/09).

### Cells and viruses

Vole embryonic fibroblasts (VEFs) were obtained from bank vole (*Myodes glareolus*) fetuses. Fetuses were obtained at around day 14 of gestation. Head and liver were carefully removed, and then the tissues were minced, treated with trypsin, and then dissolved into single cell suspension. VEFs, pooled from 4 fetuses, were cultured in MEM supplemented with 20% fetal bovine serum (FBS), non-essential amino acids, penicillin and streptomycin (Invitrogen, Carlsbad, CA, USA). Vero E6 cells (African green monkey kidney cells, ATCC; VERO C1008) were grown in Dulbecco's modified Eagle medium (DMEM) supplemented with 5% FBS, HEPES, penicillin and streptomycin (Invitrogen).

Wild type PUUV strain Kazan (PUUV-wt) [14], Vero E6 cell line-adapted PUUV strain Kazan (PUUV Kazan-E6) [14] and Umeå/305/human/95 (PUUV Umeå) [15], TBEV strain 93–783 [16], CPXV strain ATCC VR 302, LV strain 145SLG [17], and green fluorescent protein-expressing Newcastle disease virus (NDV-GFP) [18] were used in the present study. Medium used for virus infections was HBSS supplemented with 2% FBS, HEPES, penicillin and streptomycin (Invitrogen). Titration of cell-line derived hantaviruses in supernatants from infected VEFs was performed as earlier described [19]. Titration of CPXV, LV and TBEV was performed by infection of Vero E6 cells with serial dilutions of supernatants from the infected VEFs. For CPXV, plaques were counted manually at 72 hours after the infection. For quantification of LV and TBEV, at 12 days after the infection, the Vero E6 cells were fixed in 80% acetone for one hour at −20°C and rabbit anti-LV serum or rabbit anti-TBEV serum, followed by FITC-conjugated goat anti-rabbit IgG (Sigma-Aldrich) were used for detection of infected cells, respectively.

### Stimulation of innate immune responses

Poly(I∶C) (Sigma) was transfected into subconfluent VEFs using FuGene (Roche Diagnostics, Mannheim, Germany) according to the manufacturer's protocol. The amount of transfected poly(I∶C) was 25 µg per 25 cm^2^ of cells. Seventeen hours after transfection, the medium was collected, and total cellular RNA was isolated using TriPure Isolation Reagent (Roche Diagnostics) according to the manufacturer's recommendations.

A bioassay based on GFP-expressing Newcastle disease virus (NDV-GFP) [18] was used to show the presence of bioactive IFNs secreted from the poly(I∶C)-transfected VEFs. Supernatants to be tested were added to VEFs, and cells were incubated for 24 h followed by infection with NDV-GFP. Seventeen to 20 h later, the number of cells expressing GFP was determined by fluorescence microscopy.

### Immunoflourescence

Infected cells were fixed in ice cold acetone∶methanol (1∶1) for 10 min. For detection of PUUV-infected cells convalescent human anti-PUUV serum followed by a FITC-conjugated goat anti-human IgG (Sigma-Aldrich, St Louis, MO, USA) was used. Rabbit anti-pox serum, rabbit anti-TBEV serum and rabbit anti-LV serum, followed by FITC-conjugated goat anti-rabbit IgG (Sigma-Aldrich) were used for detection of CPXV-, TBEV-, and LV-infected cells, respectively.

For detection of STAT1-phosphorylation, VEFs were treated with poly(I∶C)-conditioned medium containing bank vole IFNs for 45 min or left untreated, followed by fixation and permeabilization in ice-cold acetone-methanol (1∶1) and staining as previously described [20].

Nuclei were counterstained with 4′,6′-diamidino-2-phenylindole (DAPI) (Sigma-Aldrich).

### Griess assay

Nitric oxide (NO) reacts rapidly with oxygen to form nitrite and nitrate [21]. The production of NO was measured indirectly in cell culture supernatants by determination of the level of nitrite using the Griess assay. Supernatant samples, and sodium nitrite as a standard, were mixed with equal volumes of Griess reagents (1% sulfanilamide and 0.1% naphtylethylenediamide in 5% phosphoric acid), and the optical density at 540 nm was measured by spectrophotometry. The nitrite standard was diluted in the same medium as used for the samples.

### Synthesis of cDNA and sequencing of antiviral genes

To remove any contaminating DNA, total RNA from poly(I∶C)-transfected VEFs was treated with Turbo DNA free (Ambion, Austin, TX, USA). cDNA synthesis was performed using Superscript™ III Reverse Transcriptase (Invitrogen), random primers (Invitrogen) and RNAseOUT™ Recombinant Ribonuclease Inhibitor (Invitrogen) following the manufacturer's protocol. The cDNA was amplified by PCR using Taq polymerase (Roche Diagnostics) and primers derived from known mouse and rat IFN-β and Mx sequences. BigDye Terminator v.3.1 (Applied Biosystems, Foster city, CA, USA) was used for sequencing according to the manufacturer's instructions.

### Quantitative real-time PCR

Quantitative real-time-PCRs (Q-PCRs) for bank vole IFN-β and Mx2 were developed based on the obtained bank vole sequences. Primer Express software (Applied Biosystems) was used to design primers and probes (Table 2). A concentration of 0.9 µM of each primer and 0.2 µM probe were used, respectively. Quantification of bank vole β-actin was performed using a commercial assay for mouse β-actin (Applied Biosystems). All reactions were performed using TaqMan Universal PCR Master Mix (Applied Biosystems) according to the manufacturer's recommendations on a 7900HT sequence detection system (Applied Biosystems).

**Table 2:** Primers and probes used for quantitative RT-PCR of bank vole IFN-β and Mx2.

	Forward primer	Reverse primer	MGB probe
IFN-β	CCATCAACTACGAGGAGCTTGA	TTCAGGCATGCTGAGTTGCT	CGCACACAACGCAG
Mx2	TCATCAATGGCTGAGATCTTTCA	GATGCGGTTGTGAGCTTCCT	CATCTGAATGCCTACCGC

MGB, minor groove binding.

## Results

### VEFs are susceptible to infection with several bank vole borne viruses

**Figure 1:**
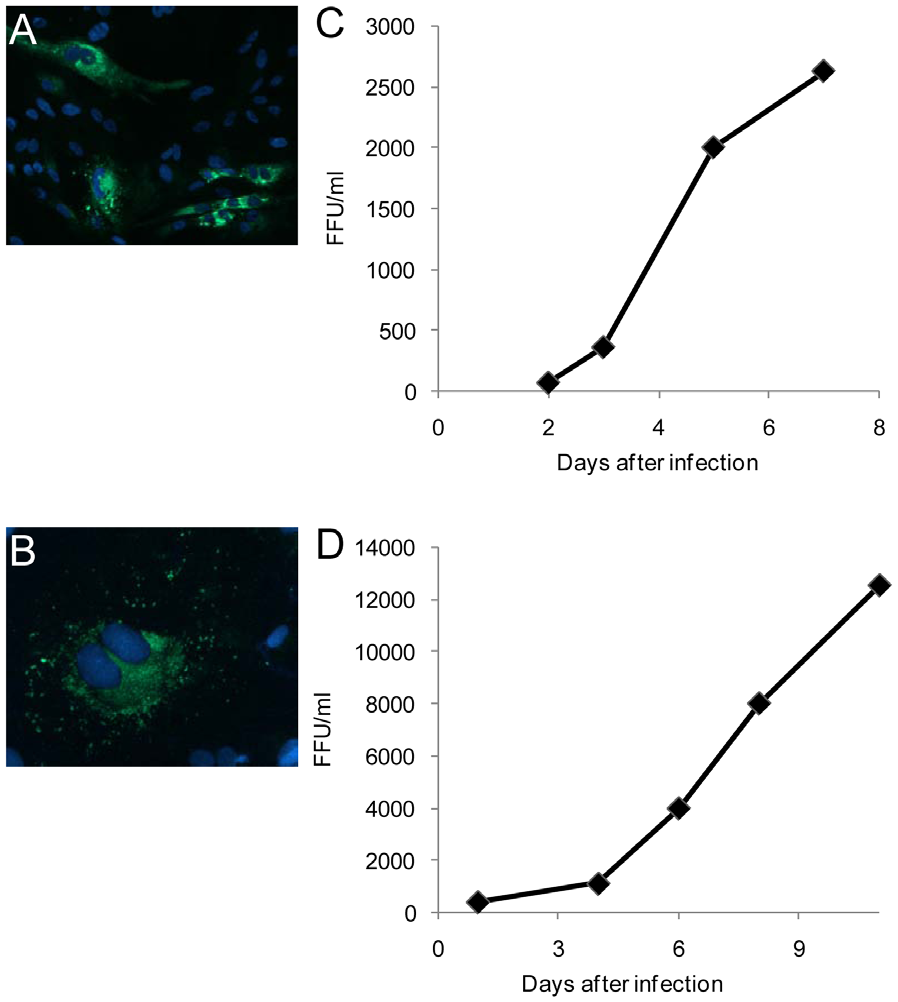
VEFs can be infected with cell-line adapted PUUV. Cells grown on coverslips were infected at a MOI of 0.1, fixed and stained (in green) for PUUV Kazan-E6 at 5 days post infection (dpi) (A), or for PUUV Umeå at 11 dpi (B). Nuclei were visualized with DAPI staining (blue). Cultivation media from VEFs infected with PUUV Kazan-E6 (C) or PUUV Umeå (D) were collected at the indicated time points post infection and the virus titre, determined as focus forming units (FFU)/ml, were assayed. Graphs represent the means from one of two or more different experiments.

In order to test if VEFs could be infected with PUUV, cells were infected with PUUV-Kazan-E6 and PUUV-Umeå and then monitored for up to 11 days. Both strains could successfully infect VEFs (Fig. 1A and 1B) and the level of progeny virus in supernatants increased steadily over time for both strains (Fig. 1C and 1D), clearly showing that VEFs are susceptible to cell-line adapted PUUV.

**Figure 2:**
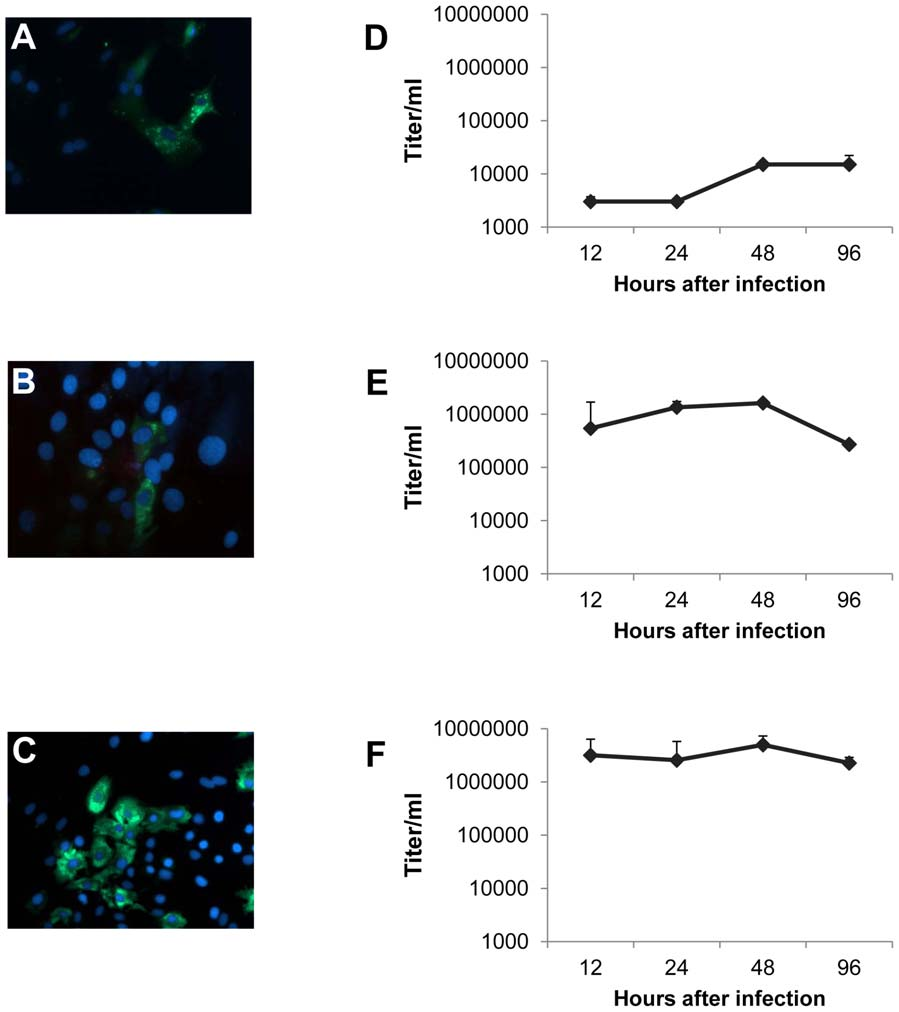
Infection of VEFs with CPXV, LV, and TBEV. Cells grown on coverslips were infected with indicated viruses at a MOI of 0.1, fixed and stained (in green) for CPXV at 17 hours post infection (hpi) (A), LV at 8 hpi (B), and for TBEV at 24 hpi (C). Nuclei were visualized with DAPI staining (blue). To measure virus production, VEFs were infected with CPXV at a MOI of 0.1 (D), LV at a MOI of 60 (E) or TBEV at a MOI of 100 (F) and cultivation media was collected at the indicated time points post infection. Virus progeny production (virus/ml cell culture medium) was determined by titration on Vero E6. Error bars represent standard deviations of the means from one experiment.

To investigate if VEFs, in addition to PUUV, are also susceptible to other viruses for which bank voles are a known reservoir, cells were next infected with CPXV, LV, and TBEV and subsequently stained for the presence of viral proteins at 17 hours post infection (hpi), 8 hpi and 24 hpi, respectively. All three viruses were shown to infect VEFs (Fig. 2A–C).

In addition, supernatants from infected VEFs were titrated on Vero E6 cells in order to analyze progeny virus production at 12, 24, 48 and 96 hpi. For CPXV, viral titers in supernatants increased from 48 hours after infection (Fig. 2D), while LV-infected (Fig. 2E) and TBEV-infected (Fig. 2F) VEFs produced a stable amount of progeny virus over time.

For CPXV and LV, a mild cytopathic effect could be observed already at 12 hpi, increasing with time; at 48 hpi and 96 hpi, very few cells remained viable for LV and CPXV, respectively. For PUUV and TBEV, no cytopathogenicity was observed (data not shown).

These results suggest that VEFs might be a suitable *in vitro*-model for studies of several zoonotic viruses associated with bank voles.

### VEFs are susceptible to infection with wild-type PUUV

**Figure 3:**
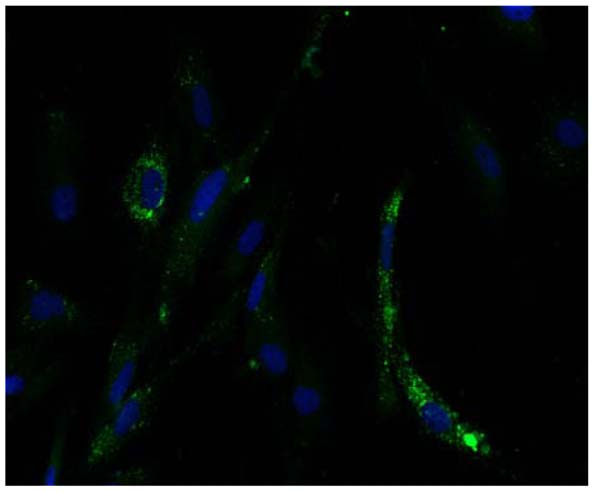
VEFs can be infected with wild-type PUUV. Cells grown on coverslips were infected with PUUV-wt, fixed and stained (in green) for PUUV-wt at 4 dpi. Nuclei were visualized with DAPI staining (blue).

In contrast to infection with cell-line adapted hantaviruses, *in vitro*-infection with wild-type virus has earlier not been reported. To test if VEFs were susceptible also for wild-type PUUV (not passaged *in vitro*), a lung sample from a bank vole experimentally infected with PUUV-Kazan-wt [22] was grinded in medium and used for infection. VEFs exposed to this suspension showed evident signs of PUUV infection (Fig. 3), showing that wild-type PUUV can successfully infect VEFs.

### Partial sequencing of innate genes, and development of Q-PCR for analyses of IFN-β and IFN-induced antiviral responses

**Figure 4:**
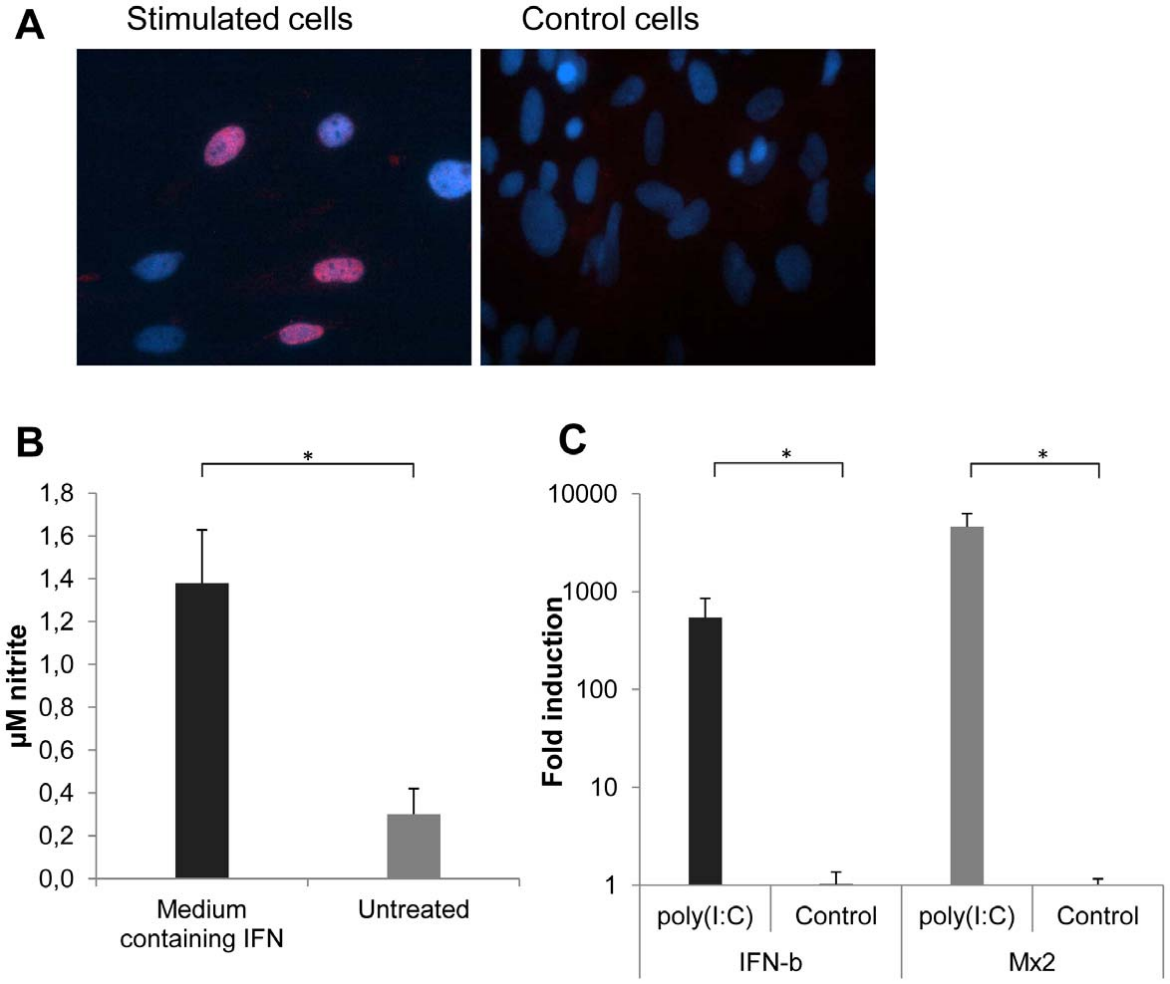
Innate immune responses in VEFs. (A) VEFs stimulated for 45 minutes with Poly(I∶C)-conditioned medium containing IFNs (stimulated cells), or with normal medium alone (control cells), were stained for phosphorylated STAT1 (red) and nuclei (blue). (B) Level of NO release in supernatants from VEFs stimulated with Poly(I∶C)-conditioned medium containing IFNs, or with normal control medium alone, for 17 hours as determined by a Greiss ELISA. Error bars represent standard deviations of the means from two experiments. (C) Transfection of VEFs with poly(I∶C) induces expression of IFN-β and Mx2 mRNA. VEFs were transfected with poly(I∶C) for 17 hours and then cellular RNA was analyzed for levels of mRNA by Q-PCR. The data were normalized using β-actin and are presented as relative expression compared to non-treated cells. Error bars represent standard deviations of the means from three experiments. *, P<0.05, Fischer exact test.

Currently there are no reagents available for specific analyses of bank vole proteins or mRNA. In order to use the VEFs as a model system for studying innate immune responses induced in cells upon viral infection, we first determined if these cells responded to stimuli that normally induce IFN production and secretion. VEFs were transfected with poly(I∶C), a double-stranded RNA analogue that can induce both type I and III IFN via the PRRs TLR3 and MDA5, and the poly(I∶C)-conditioned supernatant was then collected. At present, there are no methods available for a specific detection of bank vole IFNs, and thus a NDV-GFP bioassay [18] was used in order to detect the presence of bioactive IFNs in the supernatant. NDV is sensitive to IFNs, and cells pre-treated with supernatants that contain IFNs will consequently resist infection, while pre-treatment with supernatants that do not contain IFNs will allow NDV-infection. Pre-treatment of VEFs with supernatants from poly(I∶C)-transfected VEFs completely inhibited NDV-GFP replication, while NDV-GFP readily infected VEFs pre-treated with normal medium (data not shown). Furthermore, phosphorylation and nuclear translocation of STAT1 (Fig. 4A), and production of NO (Fig. 4B), were observed after treatment of VEFs with poly(I∶C)-conditioned supernatants. In summary this shows that innate immune responses, including the production of and response to IFNs, are activated as expected in poly(I∶C)-stimulated VEFs.

IFN-β is normally the main type of IFN produced by fibroblasts, and the Mx-proteins are sensitive markers of IFN-induced activation of antiviral responses as Mx-proteins are solely induced by IFN-α/β/λ [23]. In order to enable a quantification of IFN-β and Mx gene expression in VEFs, we set out to establish Real-Time PCR protocols for these genes. To induce IFN-β and thereby also Mx-proteins, VEFs were again transfected with poly(I∶C) and total cellular mRNA was extracted 17 h post transfection. cDNA was then synthesized, followed by PCR amplification and sequencing. Partial sequences were obtained for bank vole IFN-β and Mx2 genes (GenBank accession numbers; HQ650821 for bank vole IFN-β nucleotides 1–233, HQ650822 for bank vole Mx2, corresponding to nucleotides 217–695 in *mus musculus* Mx2, HQ650823 for bank vole Mx2 corresponding to nucleotides 747–1198 in *mus musculus*, HQ650824 for bank vole Mx2 corresponding to nucleotides 1568–2023 in *mus musculus* Mx2 ). Based on these sequences, specific TaqMan Q-PCRs protocols were designed (Table 2). As expected, analyses employing these Q-PCR protocols showed that expression of IFN-β and Mx2 genes was significantly upregulated in poly(I∶C)-transfected VEFs as compared to in mock-transfected VEFs (Fig. 4C).

### Antiviral responses in VEFs

In order to study the antiviral response against these viruses in VEFs, cells were infected with CPXV, LV, PUUV-Kazan-E6 and TBEV, and levels of mRNA expression for IFN-β and Mx2 were analyzed with Q-PCR at 6, 12, 24, 48 and 96 hours after infection.

**Figure 5:**
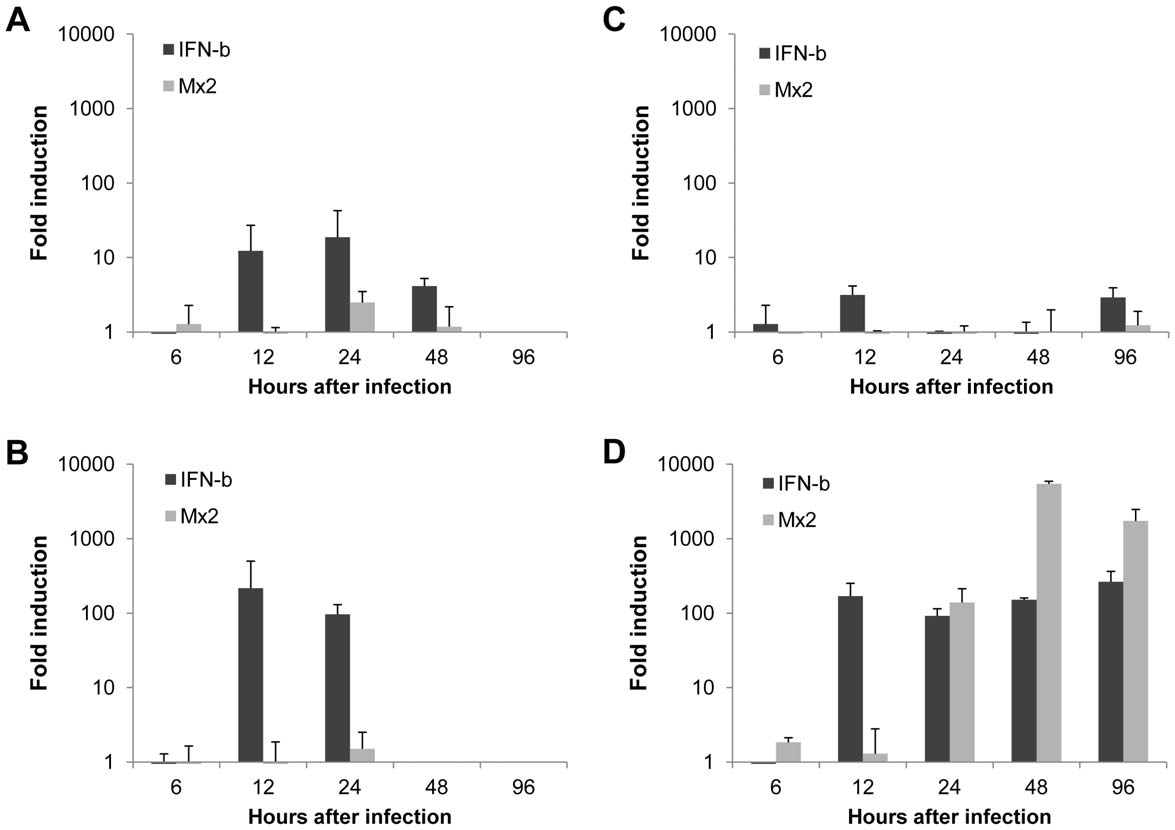
Antiviral innate immune responses in VEFs. VEFs were infected with CPXV at a MOI of 0.1 (A), LV at a MOI of 60 (B), PUUV at a MOI of 2 (C) or TBEV at a MOI of 100 (D) and cells were collected at 6, 12, 24, 48 and 96 hpi. mRNA expression for IFN-β and Mx2 was evaluated by Q-PCR. Due to lyzis of infected cells, IFN-β and Mx2 expression levels could not be defined at 96 hpi for CPXV and from 48 hpi and onwards for LV. The data were normalized using β-actin and are presented as relative expression compared to the uninfected control. Error bars represent standard deviations of the means from one experiment.

Both CPXV and LV induced IFN-β but not Mx2 from 12 hpi an onwards (Fig. 5A–B), suggesting that these two viruses activate IFN-β but can inhibit activation of an IFN-induced antiviral state in infected bank vole cells. We could not detect any clear up regulation of IFN-β or Mx2 mRNA for PUUV (Fig. 5C). In contrast, TBEV strongly induced IFN-β from 12 hpi, followed by MxA that was induced at 24 hpi and peaking at 48 hpi (Fig. 5D).

Together with the results for poly(I∶C)-transfected VEFs, these results further strengthen the value of using these cells as a model system for fundamental studies of cellular innate immune regulation against viral infections in bank voles and/or mechanisms of viral persistence.

## Discussion

We here report that VEFs, isolated from bank vole embryos, are susceptible to infection with PUUV, and also to other bank vole borne viruses like TBEV, CPXV and LV, thereby providing the first *in vitro*-model for experimental studies of viruses having bank voles as a natural reservoir. Importantly, in addition to infection of VEFs with cell line adapted PUUV, successful infection was also established with wild-type PUUV. The bank vole IFN-β and Mx2 genes were partially sequenced, and Q-PCR protocols for quantification of gene expression were developed.

Using these newly developed Q-PCR protocols, levels of IFN-β and Mx2 mRNA were analyzed in response to infection with CPXV, LV, PUUV-Kazan-E6 or TBEV. For CPXV and LV, lytic infections were observed in combination with increased IFN-β but not Mx2 expression from12 hpi and onwards. For CPXV, viral titers in supernatants increased dramatically 48 hpi at the same time point as clear cytopathic effects were observed, indicating an inefficient control of the infection in these cells. Although displaying a lytic infection, production of viral LV progeny did not increase with increased amounts of lyzed cells, indicating a less efficient replication than for CPXV. Natural infections of bank voles with CPXV and LV are believed to occur without symptoms. If these infections also cause lytic infections of cells in the natural host *in vivo* remains to be shown.

Interestingly, PUUV replicated efficiently, showing increased production of progeny virus over time, without clearly inducing IFN-β or Mx2. This suggests a strong PUUV-mediated repression of viral recognition and/or inhibition of IFN-activation in these cells. In contrast infection of human fibroblasts with the same PUUV-stock (in that study referred to as PUUV-Pa) resulted in induction of IFN-β and MxA, and inhibition of progeny virus production over time [24], suggesting that PUUV regulate bank vole cells in a different manner than human cells.

In contrast to what was observed for PUUV, both IFN-β and Mx2 were clearly induced in TBEV-infected VEFs, and virus production did not increase over time. Hence, these results indicate an innate immune reaction that keeps TBEV in check, although not strong enough to completely inhibit progeny virus production.

This *in vitro* model together with our initial characterizations will simplify future studies addressing virus-host interactions between bank vole borne viruses and natural host-derived cells, including possible regulatory effects on innate immune activation (induction of IFN-β) as well as on antiviral responses (induction of Mx2). The model presented here could easily be extended to other natural host species for hantaviruses, and hence facilitate future investigations aiming at understanding how hantavirus regulation of natural host cells differs from that of human cells, and might also provide a reliable method for isolation of wild-type viruses. The genetic diversity within bank voles might lead to differences in outcome of infection. We believe that isolations of VEFs from genetically different bank voles isolated in different geographical localities, can serve as a useful tool for studies addressing the effect of genetic differences on innate immune responses and other markers of infection, which might lead to a better understanding of bank vole-borne virus pathogenesis. Further, information on the role of infected fibroblasts during viral infections is not available and hopefully the VEF system presented here provide new knowledge about infections of bank vole cells in general. Experimental infection of natural hosts with hantaviruses have earlier been established for Sin Nombre virus (SNV), Seoul virus and PUUV [14], [25]–[27]. In addition, genes encoding deer mouse chemokines and cytokines have been sequenced [28], [29], thereby enabling studies of immune responses during SNV-infection of the natural host *in vivo*. However, to our knowledge this is the first report of an *in vitro*-model based on cells from a natural hantavirus host.

It is not known why hantavirus infection in humans causes disease, while the infections in natural hosts are asymptomatic. *In vitro*-studies performed in human cells have shown that hantaviruses can interfere with several signalling pathways that are involved in innate immune activation and function [9], but it is still unknown if these effects are different from those in the natural hosts. Investigation and comparison of the mechanisms by which hantaviruses affect human and natural host cells is important in order to better understand why hantaviruses cause disease in man and also how they establish a chronic infection in their natural hosts.

Our results suggest that some of the immune responses that are evoked in humans during hantavirus infection are suppressed in rodent reservoirs. If these differences are significant for observed differences between the asymptomatic, persistent infection in bank voles [10], compared to the transient but symptomatic infection in humans, remains to be investigated.The finding that PUUV-wt efficiently infects VEFs suggests that VEFs might serve as a tool for isolation of new PUUV strains, either from bank voles or from patients. Hantaviruses are notoriously difficult to isolate *in vitro* and therefore there is only a very limited number of cell line adapted hantaviruses available for research, which clearly hampers studies on hantavirus pathogenesis. Why infection of human cells *in vitro* with wild-type hantaviruses is normally not observed, and why it is difficult to adapt hantaviruses to growth in established cell lines, is currently not known. As of today, cell line adaptation of hantavirus is often performed on Vero E6 cells that lack the capacity to produce IFN-α/β [30], which may allow for evolution of viral substrains with phenotypical properties that differ from those of the parental wild-type strain. This has been shown for PUUV, for which cell culture adaptation is associated with the introduction of mutations [14], [31] and evolution of substrains with different phenotypes [24]. Importantly, mutations of the viral genome during cell line adaptation have also been observed for other bank vole-borne zoonotic viruses, e.g. TBEV [32] and LV [33]–[35]. It remains to be shown if selections observed during cell line adaption in non-host cell lines, like Vero E6, also occur in cells derived from bank voles.

Reverse genetics is an attractive method for production of viruses. This method allows for the production of wild-type as well as of genetically altered forms of the virus in question. However, as of today, all attempts to create a reverse genetics system for hantaviruses have failed. Interestingly, embryonic cells from the fruit bat *Rousettus aegyptiacus*, a presumed natural host for certain filoviruses, have been isolated [36], and successfully infected with Ebola and Marburg viruses. Interestingly, these cells have enabled rescue of Marburg virus by use of reverse genetics [37]. Possibly, embryonic cells from hantavirus natural hosts might contribute to a reverse genetics system also for hantaviruses.

In conclusion, we show that VEFs can serve as a tool for studies of several known bank vole borne viruses, including important human pathogens. Using this method, it will hopefully be possible to better understand how these zoonotic viruses interfere with host cell signaling pathways and how they affect induction of innate immune responses.
